# Abandoning sex: multiple origins of asexuality in the ciliate *Tetrahymena*

**DOI:** 10.1186/1471-2148-14-112

**Published:** 2014-05-28

**Authors:** F Paul Doerder

**Affiliations:** 1 Department of Biological, Geological and Environmental Sciences, Cleveland State University, 2121 Euclid Avenue, Cleveland, OH 44115, USA

**Keywords:** Asexual, Amicronucleate, Natural populations, Barcode, Putative new species, Evolution, Muller’s ratchet, Macronuclear assortment

## Abstract

**Background:**

By segregating somatic and germinal functions into large, compound macronuclei and small diploid micronuclei, respectively, ciliates can explore sexuality in ways other eukaryotes cannot. Sex, for instance, is not for reproduction but for nuclear replacement in the two cells temporarily joined in conjugation. With equal contributions from both conjugants, there is no cost of sex which theory predicts should favor asexuality. Yet ciliate asexuality is rare. The exceptional *Tetrahymena* has abandoned sex through loss of the micronucleus; its amicronucleates are abundant in nature where they reproduce by binary fission but never form conjugating pairs. A possible reason for their abundance is that the T*etrahymena* macronucleus does not accumulate mutations as proposed by Muller’s ratchet. As such, *Tetrahymena* amicronucleates have the potential to be very old. This study used cytochrome oxidase-1 barcodes to determine the phylogenetic origin and relative age of amicronucleates isolated from nature.

**Results:**

Amicronucleates constituted 25% of *Tetrahymena*-like wild isolates. Of the 244 amicronucleates examined for *cox*1 barcodes, 237 belonged to *Tetrahymena*, seven to other genera. Sixty percent originated from 12 named species or barcoded strains, including the model *Tetrahymena thermophila*, while the remaining 40% represent 19 putative new species, eight of which have micronucleate counterparts and 11 of which are known only as amicronucleates. In some instances, *cox*1 haplotypes were shared among micronucleate and amicronucleates collected from the same source. Phylogenetic analysis showed that most amicronucleates belong to the “borealis” clade in which mating type is determined by gene rearrangement. Some amicronucleate species were clustered on the SSU phylogenetic tree and had longer branch lengths, indicating more ancient origin.

**Conclusions:**

Naturally occurring *Tetrahymena* amicronucleates have multiple origins, arising from numerous species. Likely many more new species remain to be discovered. Shared haplotypes indicate that some are of contemporary origin, while phylogeny indicates that others may be millions of years old. The apparent success of amicronucleate *Tetrahymena* may be because macronuclear assortment and recombination allow them to avoid Muller’s ratchet, incorporate beneficial mutations, and evolve independently of sex. The inability of amicronucleates to mate may be the result of error(s) in mating type gene rearrangement.

## Background

Asexuality is rare in ciliates, reported for only a few genera [1], but is exceptionally common in *Tetrahymena* wild isolates [2,3]. This is likely a consequence of ciliate nuclear dimorphism and the peculiarities of the *Tetrahymena* macronucleus which allow it to evolve independently of sex. Like all ciliates, *Tetrahymena* possess a germinal micronucleus and a somatic macronucleus (reviewed in [4]). The micronucleus divides by mitosis and meiosis and forms gametic nuclei during conjugation, the sexual phase of the life cycle; it provides genetic continuity between generations and is transcribed only during conjugation. The larger, compound macronucleus is transcriptionally active and controls the cell’s phenotype. During conjugation, a temporary union between two cells, the conjugants acquire new micronuclei through recombination and reciprocal fertilization, and in the process become genetically identical. They also replace their macronuclei with new ones derived from zygotic micronuclei. After separation, the two exconjugants reproduce by binary fission. In many ciliates there is a period of sexual immaturity during which cells cannot mate. This paper concerns the multiple origins of the numerous asexual *Tetrahymena* encountered in nature. Asexual *Tetrahymena* lack the micronucleus and thus are asexual by definition; they cannot form gametic nuclei required for fertilization. Paradoxically, *Tetrahymena* amicronucleates also cannot conjugate, a function controlled by the macronucleus.

Much of the theory associated with eukaryote sexuality does not apply to ciliates. For instance, in animals, parthenogenetic females producing only daughters waste no resources on males, the so-called two-fold cost of sex. By this argument, asexuality should be more common. Yet, sex is rarely abandoned in animals and plants, and when it is, with notable exceptions, it is evolutionarily unstable [5]. The frequently cited reason for the persistence of sex is the benefit provided by new gene combinations afforded by meiosis and the fusion of gametes. Ciliates, however, do not have males, and therefore no such two-fold cost of sex; nor do related arguments based on the costs of anisogamy and allocation of parental resources apply. The two ciliate conjugants are equal partners and both acquire the same genotype at the moment of fertilization. Yet, as noted, with the major exception of *Tetrahymena*, asexuality is rare in ciliates. Moreover, it has been argued that many, if not all, purportedly asexual micronucleate ciliates are in fact sexual, albeit “secretively” [6]. Another form of the argument for the persistence of sex is Muller’s ratchet [7], which postulates that in asexual lineages the genome is effectively a single, non-recombining linkage group in which the accumulation of deleterious mutations results in lineage extinction. Sex persists because recombination not only generates genetic diversity, it breaks up combinations of deleterious alleles. Ciliates also appear to benefit from sex. Because the micronuclear genome is not expressed until conjugation, its genes are immune from selection and mutations can accumulate. Indeed, it is well documented that micronuclei age and eventually lose the ability to transmit genes [8-10]. As in other systems, meiosis effects repair of genetic damage [11], up to a limit. Ciliates also likely benefit by replacing the macronucleus, as there is an old and extensive literature on macronuclear failure and death of clones prevented from having sex [8]. The exception is the ciliate *Tetrahymena* which appears to be capable of unlimited division. While Muller’s ratchet applies to its micronucleus, the ratchet appears not to apply to its macronucleus (see below).

Long studied in the laboratory [12], *Tetrahymena* amicronucleates account for 30–50% of isolates in some collections [2,3]. Moreover, none of them have been observed to conjugate. Were they to mate, even secretively, studies suggest that such “sex” either would be lethal [13] or would result in the acquisition by the amicronucleate of a micronucleus that then would allow true sex [14]. It appears that *Tetrahymena* really do abandon sex, particularly in natural populations. With one exception [13], amicronucleates formed in the laboratory die. This includes spontaneous amicronucleates formed in hypodiploid cells [15] as well as those formed by experimental means [1,16]. In both cases oral abnormalities are present, suggesting that the micronucleus has an essential somatic function [1,16,17] even though micronuclear transcription is undetected except at conjugation. Wild *Tetrahymena* amicronucleates are unable to form conjugating pairs despite the fact that mating type expression is a macronuclear, not micronuclear, function. Mating type determines sexual compatibility for conjugation and is fixed during macronuclear development [18] either by inherited genotype or by extensive rearrangement of a complex mating type gene transmitted by the micronucleus [19]. For the latter, without proper rearrangement, the mating type genes cannot be expressed. The inability of amicronucleates to mate suggests that in addition to the loss of the micronucleus, amicronucleates have defective mating type genes, or that they are permanently immature.

The exceptional occurrence of asexuality in natural populations of *Tetrahymena* almost certainly involves macronuclear assortment, a phenomenon absent in most other ciliates. Initiated at the first division of the macronucleus following conjugation, assortment refers to the production during binary fission of lineages (aka subclones) that irreversibly express one phenotype or the other; all that is needed is genetic polymorphism. Thus a heterozygous cell, for *any* locus, will, through repeated fissions, yield assortant subclones which have one allele or the other, but not both; the unexpressed allele is indeed lost. The units of assortment are the ~45 copies of the 180 macronuclear chromosomes [20]. Assortment means that Muller’s ratchet [7] probably does not apply to *Tetrahymena* macronuclei. There are two considerations. First, unlike in plant and animal asexuals, the 180 macronuclear chromosomes do not function as a single, fixed linkage group during binary fission. Instead, these chromosomes literally assort independently at each macronuclear division, potentially forming numerous combinations of genes. Moreover, though genes on the same chromosome usually coassort, because there are ~45 copies, they may recombine within the macronucleus at binary fission [21]. Second, as assortment proceeds it produces subclones that contain different number of alleles. For instance, should a cell inherit a new mutation occurring in one of the ~45 copies of a macronuclear chromosome, that cell would give rise either to daughters with one copy each of the new mutation or to daughters with two and zero copies; the other 43–45 alleles would be wildtype. As with genetic drift, assortment could fix the new allele. However, when a new *deleterious* mutation arises in one of the 45 copies of a macronuclear chromosome, it will be eliminated by selection against the subclones that contain copies of the mutant allele, favoring subclones that have lost that allele due to assortment. Because of these two considerations, it is highly unlikely that neither a single deleterious allele nor combinations of deleterious alleles will become fixed, considerably delaying Muller’s ratchet, if indeed it applies at all. By eliminating deleterious mutations and accumulating advantageous ones by assortment, a macronuclear lineage can recombine and test new combinations of genes in different subclones, all in the absence of sex or any contact between cells. Though assorted combinations of genes are erased at conjugation in micronucleate lineages, assortment continues in asexual lineages. In essence, macronuclear assortment means that amicronucleates can evolve independently of their sexual counterparts. Some amicronucleates therefore may be very old.

The high frequency of wild *Tetrahymena* amicronucleates and the possibility of evolution independent of sex raise questions about their origin. Are they derived from few or many micronucleate species? Are they of contemporary origin or are they ancient? Because amicronucleate tetrahymenas do not mate, they cannot be associated with progenitor micronucleate species by standard mating tests. The species origin of amicronucleates was first explored when molecular methods were developed to identify species without the use of mating reactions. Using differences in isozyme mobilities, Borden [22] grouped amicronucleate strains of what was then called “T. pyriformis” into five phenosets (A-E). None shared mobilities with micronucleate species. Subsequently, Nanney and McCoy [23] named four of these classical amicronucleates as *T. pyriformis* (phenoset A), *T. elliotti* (phenoset B), *T. furgasoni* (phenoset C), and *T. lwoffi* (phenoset E). Later, *T. lwoffi*, was withdrawn as synonymous with *T. furgasoni*[24], a move consistent with *cox*1 (mitochondrial cytochrome oxidase I) and SSU (nuclear small ribosomal subunit RNA) sequences [25]. The criterion for naming these amicronucleates species was that they differed in isozyme mobilities as much as micronucleate species differed among themselves. Based on sequences of the D2 segment of the LSU (nuclear large ribosomal subunit RNA) Preparata et al. [26] associated some wild amicronucleates with micronucleate species *T. elliotti*, *T. borealis*, and *T. tropicalis* but left unresolved the origin of numerous other amicronucleates.

This study uses *cox*1 barcodes [25] to determine the origin of 244 *Tetrahymena*-like amicronucleates obtained from nature. Sixty percent of the isolates are associated with named species, including *T. thermophila*, while the remaining 40% are distributed among putative new species, many without micronuclear counterparts. A model for the origin of amicronucleates is presented and the possibility of evolution without sex is further discussed.

## Methods

Wild cells were collected as described by Doerder and Brunk [27] as part of a larger study on the biogeography of *Tetrahymena*. Following isolation as clonal populations, isolates were cultured either in Cerophyll inoculated with *Klebsiella pneumoniae* or in axenic PPY (1% proteose peptone, 0.15% yeast extract, 0.001 M FeCl_3_). In most instances, to identify *T. thermophila*, bacterized cells were challenged with testers of all seven of its mating types. Cells which did not mate with *T. thermophila* testers were either sexually immature *T. thermophila*, amicronucleate, or another species. The presence/absence of the micronucleus was determined by fluorescence microscopy of (usually bacterized) cells vitally stained with acridine orange. In some instances, conjugants, all of which had micronuclei, were observed.

DNA from Cerophyll or PPY grown cells was purified with a modified microwave procedure [28] as previously described [29]. The *cox*1 (mitochondrial cytochrome oxidase I) barcode region was amplified in standard PCR with primers *cox*1ATf (ATGTGAGTTGATTTTATAGA) and 689r (CTCTTCTATGTCTTAAACCAGGCA), purified with shrimp alkaline phosphatase and exonuclease III, and sequenced in both directions with the same primers. The SSU (nuclear small ribosomal subunit RNA) was similarly amplified and sequenced with primers SSUf (TTACATGGATAACCGAGCTA) and SSUr (GCAGGTTCACCTACAGATAC). The D2 region (190 bp) of the LSU was amplified with D2f (AAGGGAGATTTCAAAGAGTCG) and D2r (CTACGAGCTTCCACCAGAGT). A one kilobase pair region of the actin gene was amplified with ACTAT (ATGGCTGAAAGTGAATCCC) and ACT1K (CTCAGGAGGAGCAACAAC). Sequences were assembled, edited and aligned with Geneious Versions 5 and 6 created by Biomatters at http://www.geneious.com/. NJ trees were drawn and edited with Mega 5.0 [30]. Networks were drawn by Network and Network Publisher (fluxus-engineering.com) [31,32].

The mitochondrial *cox*1barcodes reported by Chantangsi et al. [25] and Kher et al. [33] are better able to discriminate among *Tetrahymena* species than nuclear SSU sequences. Using the extensive collection of *Tetrahymena* species in the American Type Culture Collection these authors defined a 689 nucleotide *cox*1 barcode and provided ~1600–2300 SSU nucleotide sequences for most named *Tetrahymena* species. Kher et al. [33] also showed that unknowns can, in most instances, be identified by the *cox*1 barcodes. The *cox*1 sequence of the type strain (Table 1) designated by Chantangsi et al. [25] and Kher et al*.*[33] was used for species identification. Arbitrarily, *cox*1 differences of >4% were used to designate an unknown as a putative new species. Using 36 *Tetrahymena* species and six unnamed isolates, Chantangsi et al. [25] found an average *cox*1 difference of 10.5% ± 0.74 (SEM), with a range of 0% to over 15%. Among 990 pairwise comparisons, only 17 were <2%, and many of these were suspected to be the result of strain misidentification or mislabeling. In the larger project of which the present paper is a part, *cox*1 differences among valid species range from 2.5% between *T. americanis* and *T. hegewischi* to >20% between *T. paravorax* and several other species. The intraspecific difference is 0–3.5% (*T. thermophila* has the largest intraspecific range), with most species <2%. The choice of 4% as a cutoff for putative species represents a compromise between false positives and false negatives. It also accommodates some members of the “americanis” clade which have *cox*1 differences of 5–6%.

**Table 1:** Wild amicronucleates among named species

	**Number**			**Number wild *cox*** **1 haplotypes**				
**Species**	**Wild isolates**	**Mic**	**Amic**	**Mic**	**Amic**	**Shared**	**% amic *cox*** **1 identity to type**	**ATCC type strain**
*T. borealis*	62	25	37	2	8	5	98.5-100	30317
*T. canadensis*	22	16	6	7	5	1	99.1-99.6	30368
*T. elliotti*	87	49	35	10	8	5		nd^a^
*T. furgasoni*	1	0	1	na	1	na	99.2	30006
*T. mobilis*	6	0	6	na	4	na	99.1-99.9	CCAP1630/22^b^
*T. pyriformis*	13	0	13	na	4	na	99.7-99.9	30327
*T. shanghaiensis*	1	0	1	na	1	na	99.2	205039
*T. thermophila*	10709	147	8	15	4	1	98.0-100	SB210^c^
*T. tropicalis*	9	1	8	na	7	1	96.4-97.1	30276
*T. vorax*	28	0	28	na	2	na	99.9-100	30421
NI/SSU/RA/CO	4	0	4	na	4	na	na	nd

Mic, possessing a micronucleus.

Amic, not possessing a micronucleus.

na, not applicable.

ATTC, American Type Culture Collection, Bethesda, MD.

^a^Type strain not designated.

^b^CCAP, Culture Collection of Algae and Protists, Scotland, UK.

^c^SB210, [GenBank:AF396436].

SSU and *cox*1 sequences of wild isolates used in this paper are deposited with accession numbers [GenBank: KJ028477-KJ028745]. Species identification and collecting information for isolates are included as Additional file 1.

## Results

### Amicronucleate frequencies and species

**Figure 1:**
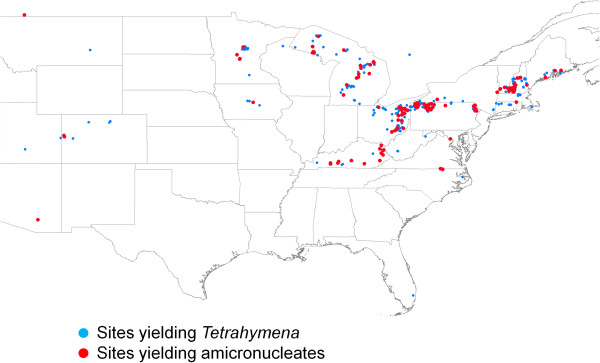
**Map of locations where *Tetrahymena *** **(blue and red) and amicronucleates (red) were collected.**

**Figure 2:**
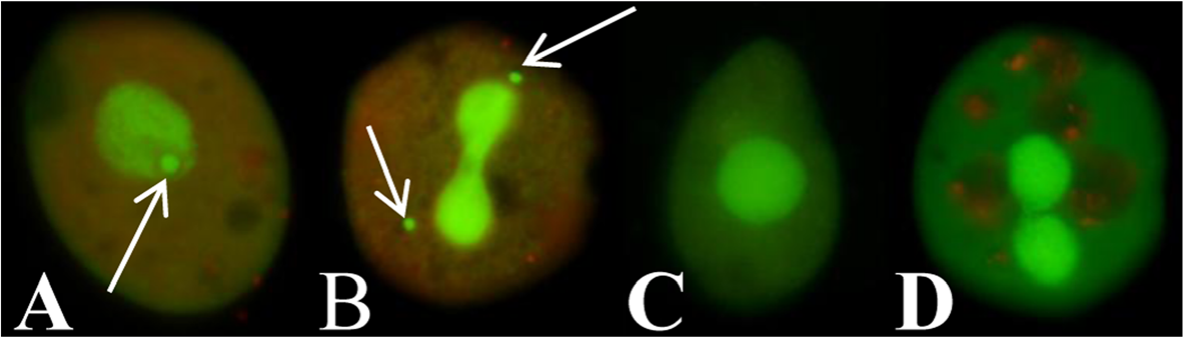
**Acridine orange vital staining of micronucleate and amicronucleate cells. (A)** Non-dividing cell with single macronucleus and a micronucleus (arrow). **(B)** Dividing cell with post-mitotic micronuclei (arrows). **(C)** Non-dividing amicronucleate cell. **(D)** Dividing amicronucleate cell. Distortion in dying cells **(A,B,D)** from typical pyriform shape **(C)** is normal with acridine orange and enhances visualization of micronuclei.

Of 2609 *Tetrahymena*-like wild isolates collected since 2006, primarily in the northeast quadrant of the US (Figure 1) as part of a larger project on the biogeography of *Tetrahymena*, 25.4% were amicronucleate (Figure 2). The occurrence of amicronucleates in individual collections (obviously dependent on sample size) varied from zero to 100%, confirming with the “high numbers” of 30–50% reported by others [2,3]. Among 387 collecting sites from which *Tetrahymena*-like isolates were examined for micronuclear presence, 173 (44.5%) contained amicronucleates, 337 (87.0%) contained micronucleates, and 123 (31.8%) contained both micronucleate and amicronucleate isolates. In addition to their abundance, amicronucleates, like micronucleates, were broadly distributed (Figure 1).

To determine the species origin of these amicronucleates, 244 isolates were examined for the *cox*1 barcode (Tables 1 and 2). Tables 1 and 2 also include totals of relevant micronucleate isolates. Of the amicronucleates, 147 (60.2%) were assigned to 11 named *Tetrahymena* species or previously barcoded strains [25] (Table 1), and two were assigned to *Glaucoma chattoni*, a species in a genus closely related to *Tetrahymena*. Consistent with past observations [26] the *cox*1 sequences assigned amicronucleates to *T. borealis*, *T. elliotti*, and *T. tropicalis*, as well as to species previously known only from micronucleate isolates (see below). The remaining 97 amicronucleate isolates differed by >4% from type strains and therefore were designated as putative new species (see Methods). Four belonged to two putative new species of *Glaucoma*, one with micronuclei, the other without. One belonged to a putative new species of *Dexiostoma* (another closely related genus) that has micronuclei. Ninety isolates were distributed among 16 putative new *Tetrahymena* species (Table 2). Of these, 10 consisted exclusively of amicronucleate isolates. In relative order of abundance, *T. borealis* and *T. elliotti* were the most frequent amicronucleates, followed by nsp7 and *T. pyriformis*. At the other extreme, two named species and four putative new species of amicronucleates were represented by single isolates. The sample of *T. vorax*, a species which preys on other tetrahymenas, is biased upward because all isolates that ate the *T. thermophila* mating type testers (see Methods) were subjected to barcode analysis and all were *T. vorax*. Nsp15 is also biased upward because the ponds containing them were repeatedly sampled for other purposes.

**Table 2:** **Amicronucleates among putative new species of *Tetrahymena***

	**Number**			**Number *cox*** **1 haplotypes**			
**New species**	**Wild isolates**	**Mic**	**Amic**	**Mic**	**Amic**	**Shared**	**Pairwise *cox*** **1 identity**
nsp7	31	4	27	2	8	2(0)^a^	99.2%
nsp9	40	36	2	4	1	1(1)	99.9%
nsp15	17	0	17	na	2	na	99.1%
nsp16	4	1	3	1	3	1(1)	98.9%
nsp18	13	4	9	1	5	1(1)	99.7%
nsp19	6	0	6	na	2	na	99.7%
nsp20	6	0	6	na	2	na	99.7%
nsp21	8	3	5	3	2	2(1)	98.4%
nsp22	4	0	4	na	1	na	100.0%
nsp23	3	0	3	na	1	na	100.0%
nsp25	2	0	2	na	2	na	99.4%
nsp31	5	2	2	2	2	1(0)	99.6%
orphans	10	6	4	na	na	na	87.0%

Mic, possessing a micronucleus.

Amic, not possessing a micronucleus.

na, not applicable.

^a^Number of collecting sites sharing a haplotype in parentheses.

Three of the classical named amicronucleates [22,23] listed in Background were found. For each species, *cox*1 sequences were highly similar to those of the type strains (Table 1). *T. elliotti*, as reported earlier [2], consisted of both micronucleate and amicronucleate forms. In 4/8 of these cases in which micronucleates and amicronucleates occurred in the same pond, the *cox*1 haplotypes were identical, suggesting that the amicronucleates recently arose in those ponds. *T. pyriformis*, first found in France [12], consisted only of amicronucleates distributed among three *cox*1 haplotypes that differed by one, two or three nucleotides. *T. furgasoni* (syn. *T. lwoffi)* was found only once. Though the type strain (Table 1) for this species is described as “GL” from Paris, France, it is almost certainly from elsewhere. The label “GL” is more appropriately applied to the type strain of *T. pyriformis*[22]. The apparent mislabeling of classical amicronucleate strains has been discussed before [22], as has the mislabeling of certain archived strains [25].

This paper adds *T. mobilis*, *T. shanghaiensis*, the unnamed NI/SU/RA/CO strains, and *T. thermophila* to the list of named species (or barcoded strains) having amicronucleates. All of the *T. mobilis* reported here are amicronucleate; the micronuclear status of the original European isolate [34] is unknown [35]. *T. shanghaiensis*, isolated as a selfing micronucleate strain [36], reportedly produces viable amicronucleates [37] capable of conjugation [38]. If verified, this would be the second exception to the long standing observation that amicronucleate tetrahymenas do not mate. The NI/SU/RA/CO unnamed species consists of several strains whose *cox*1 sequences differed by <4%. NI, RA and CO were isolated from guppies obtained from Singapore [39], whereas SU, with the most divergent *cox*1 sequence, was isolated from wet soil in CA, USA [25]. Strain NI is has a micronucleus [39]; the micronuclear status of the others is unknown. The four amicronucleate wild isolates reported here were collected from water samples. This species may have diverse ecological habitats.

**Figure 3:**
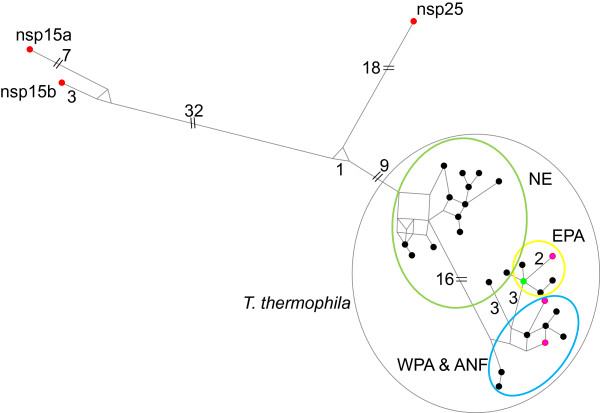
**Network diagram of micronucleate (black circles), and amicronucleate (red, new species; fuchsia, *T. thermophila*) and shared (green) *cox*1 haplotypes.** Numbers indicate number of nucleotide changes within 663 base pair region of *cox*1 barcode. Black oval, *T. thermophila*. Green oval, haplotypes in New England states (CT, NH, MA, ME, VT). Yellow oval, haplotypes in eastern PA. Blue oval, haplotypes in western PA and Allegheny National Forest of PA. Green haplotype is that of type strain SB210 and is shared by both micronucleate and amicronucleate isolates.

Though mentioned briefly elsewhere [27], this paper formally adds *T. thermophila* to the list of named species for which amicronucleates occur in nature. In nearly six decades of research, in numerous laboratories, the only example of a viable amicronucleate *T. thermophila* is “pig” discovered following mutagenesis [13]. As referenced in Background, both spontaneous amicronucleates and experimentally induced amicronucleates always die. It was therefore somewhat unexpected to find amicronucleate *T. thermophila* among natural isolates (Table 1). Five of the eight occurred in ponds with resident populations of micronucleate *T. thermophila*, two in ponds near (<2.0 km) multiple ponds with *T. thermophila*, and one in an area where no *T. thermophila* have been found despite repeated sampling. None mated either with each other or with any of the seven mating type testers despite repeated challenges over several years. The *cox*1 haplotypes differ by <2% from type strain SB210 (inbred strain B), well within the >96.4% sequence identity among *cox*1 haplotypes of micronucleate isolates. Based on identity of SSU and D2 LSU and near identity of a portion of the actin gene (Table 3), there is no doubt that these amicronucleates are *T. thermophila*. The amicronucleate *cox*1 haplotypes are consistent with their geographical origin (Figure 3). That is, those from EPA (eastern Pennsylvania) have haplotypes more closely related to micronucleate haplotypes from EPA, and those from WPA (western PA) are more closely resemble micronucleate haplotypes from nearby WPA ponds.

**Table 3:** **Properties of amicronucleates closely related to *T. thermophila***

						**Identity to *T. thermophila *** **type SB210**				
**Species**	**Location**	**# sites**	**# isolates**	**# *cox*** **1 haplotypes**	**Pairwise identity among isolates**	***cox*** **1**	**D2**	**SSU**	**actin**	**Self-splicing LSU intron**
nsp15a	WPA	2	6	1	100%	90.8%	nd^a^	100%	100%	absent
nsp15b	ANF	4	7	1	100%	92.2%	100%	100%	99.8%	absent
nsp25	NH	2	2	2	99.2%	94.8%	nd	100%	100%	present
*T. thermophila* amic	WPA	3	5	3	99.1%	99.3%	100%	100%	99.9%	present

D2, 190 nucleotide region of large ribosomal subunit RNA.

SSU, nuclear small ribosomal RNA.

WPA, western Pennsylvania.

ANF, Allegheny National Forest.

NH, New Hampshire.

^a^Not determined.

**Figure 4:**
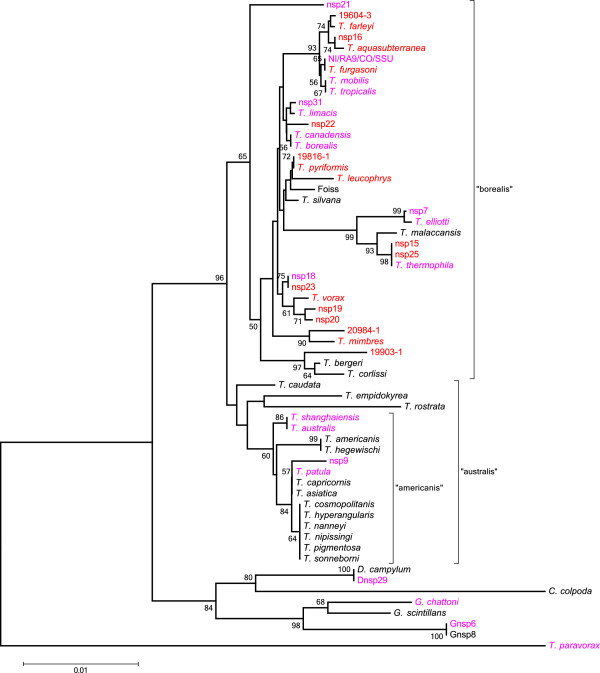
**Unrooted NJ of SSU (1384 nt region) of all named species, previously barcoded strains, and putative new amicronucleate species.** The tree was constructed using the Kimura 2-parameter methods and is drawn to scale. Bootstrap test (1000 replicas) percentages >50% are shown at nodes. Red: species known only from amicronucleate isolates. Black: species known only from micronucleate isolates. Fuchsia: species with both micronucleate and amicronucleate isolates. Genera: T., *Tetrahymena*; D., *Dexiostoma*; C., *Colpidium*; G., *Glaucoma*.

Amicronucleates nsp15 and nsp25 (Tables 2 and 3) are closely related to *T. thermophila* (Figures 3 and 4)*.* Both were collected in heavily sampled regions with numerous *T. thermophila* populations, but no micronucleates with similar haplotypes were found. Both are identical, or nearly so, to type strain SB210 with respect to SSU, D2 and actin (Table 3). The most closely related to *T. thermophila* is nsp25 (Figure 3) whose *cox*1sequence differs from type strain SB210 by 5.2%. It was found at two sites in NH separated by 21.7 km, one with resident *T. thermophila*. The other species, nsp15, was formed by pooling isolates whose 3.3% *cox*1 difference fell below the 4% threshold for designation as putative new species. These isolates were collected from two different PA locations separated by about 80 km. Those from WPA (2 sites, 2.4 km apart) shared a single haplotype, and those from ANF (Allegheny National Forest) (4 sites, maximum 15 km apart) shared a different single haplotype. Considered separately for the sake of argument (Table 3, Figure 3), nsp15a (WPA) and nsp15b (ANF) differ from *T. thermophila* SB210 by 9.2% and 7.8%, respectively. Though found only in west PA, nsp15 is more closely related to *T. thermophila* micronucleates from New England than to those from west PA (blue oval, Figure 3). As another difference, neither nsp15a nor nsp15b have the self-splicing intron [40] found in the LSU of both *T. thermophila* and nsp25. Nsp15 may be more anciently derived from *T. thermophila*.

### Amicronucleate phylogeny

The distribution of amicronucleates from this survey and the literature [35] is mapped to a SSU phylogenetic tree (Figure 4) that includes the named *Tetrahymena* species for which SSU sequences are available in GenBank [35]. Of the named *Tetrahymena* species, 17 have amicronucleates. Twelve of these have both micronucleate and amicronucleate forms. The majority of amicronucleates occur in the large “borealis” clade, with far fewer in the “australis” clade. These two clades were first identified based on the D2 region of the LSU [41] and appear to represent a major division in the genus *Tetrahymena*[25]. As discussed below, it may be relevant to the cytogenetic origin of amicronucleates that these two clades differ in the mechanism of mating type determination.

In addition to nsp15, nsp25 and *T. thermophila* described in the previous section, Figure 4 shows additional instances in which putative new *cox1* amicronucleate species have identical SSU sequences. *T. pyriformis* and amicronucleate orphan 19816-1 have *cox*1 sequences that are 95.9% identical, and nsp18 and nsp23 *cox*1 sequences are 94.4% identical. There are also instances, particularly in the “americanis” clade but also in the “borealis” clade in which named species which show no breeding affinity (i.e., *bona fide* biological species) have identical SSU sequences. There were no instances in which *cox*1 sequences were similar and SSU differed.

## Discussion

The results indicate that *Tetrahymena* species have abandoned sex numerous times, in many species, both in the past and contemporaneously. Amicronucleates comprised ~25% of wild *Tetrahymena* isolates and were found in just under half of the sites yielding *Tetrahymena*-like ciliates. *Cox*1 barcodes associated wild amicronucleates with multiple species: 12 named species (11 *Tetrahymena* and one *Glaucoma*) and 19 putative new species (17 of *Tetrahymena*, one of *Glaucoma* and one of *Dexiostoma*), nine of which have micronucleate counterparts and 10 of which are known only from the amicronucleate isolates. As summarized in Table 4, amicronucleates are now associated with ~50% of *Tetrahymena* species, with 16 named or putative new species known only as amicronucleates. In view of the limited geographic area covered in this study (Figure 1), more extensive collecting and a broader geographical scale doubtless will yield numerous additional *Tetrahymena* species with amicronucleates.

**Table 4:** **Numbers of micronucleate and amicronucleate species of *Tetrahymena***

	**Micronucleate only**	**Amicronucleate only**	**Mixed**	**ND**	**Total**
Named species/strains^a^	22	6	13	1	42
Putative new species^b^	13	10	6	–	29
Total	35	16	19	1	71

^a^Includes strain NI/SU/RA/CO, data from Lynn and Doerder [31].

^b^This study and Doerder (unpublished).

In contrast to finding putative new species of *Tetrahymena* with wild amicronucleates and adding to the list of micronucleate species with natural amicronucleates, this survey did not find micronucleate counterparts of species previously known only from amicronucleates. This includes *T. pyriformis* and *T. vorax* both of which were relatively common and found at multiple sites (biogeography details to be published elsewhere). It also includes *T. furgasoni*, though it is represented by a single isolate. Though data are limited, amicronucleate isolates tend to outnumber micronucleate isolates in the clades (Figure 4, Table 1) containing these three species. For instance, in the clade containing *T. furgasoni*, 7/8 *T. tropicalis* were amicronucleate and as were wild *T. mobilis*. All of the NI/SU/RA/CO reported here were amicronucleate. The clade containing *T. vorax* also is largely amicronucleate, the majority of nsp18 being amicronucleate. Because these clades were populated by amicronucleates independent of this survey (e.g., *T. farleyi* and *T. aquasubterranea* in the clade with *T furgasoni*), it is unlikely that the additional amicronucleates reported here are solely due to vagaries of sampling. There is no reason to believe that amicronucleates are overly abundant in the presently sampled region, as numerous amicronucleates were found in Elliott’s surveys [3]. Moreover, *T. aquasubterranea*, the only *Tetrahymena* known from the largely under sampled African continent, is an amicronucleate [42]. However, it is possible that these amicronucleates are descended from micronucleate species that are yet to be discovered or that have gone extinct.

The precise age of amicronucleates is difficult to determine, but likely ranges from contemporary to ancient. As a first approximation, it is reasonable to assume that an amicronucleate that shares a *cox*1 haplotype with a micronucleate recently arose from that micronucleate lineage. Most of the named species (Table 1) and putative new species (Table 2) with both micronucleates and amicronucleates have at least one instance of such shared haplotypes. Contemporary origin is indicated when a shared haplotype occurs in the same pond, as with several instances of *T. elliotti* and several putative new amicronucleate species*.* Less recent origin may be indicated by small *cox*1 sequence differences, as with *T. thermophila* whose amicronucleates are primarily found in areas with endemic micronucleates. Even older origin is suggested by greater sequence divergence and broader geographical distribution as might be the case for some amicronucleates of *T. elliotti* and perhaps *T. tropicalis*. It is possible that some amicronucleates are ancient, originating millions of years ago. Wright and Lynn [43] calibrated ciliate SSU sequence divergence using *Ichthyophtherius*, an obligate ectoparasite of teleost fish, and calculated that 1% difference in SSU sequences corresponds to 72–80 million years. By this criterion, the separation of “borealis” and “australis” clades occurred a maximum of 158–176 million years ago, with “borealis” radiating 4–72 million years ago. Some amicronucleates therefore could be tens of millions year old. For instance, based on the estimated radiation of the “borealis” clade, nsp16 and orphan 19604-3 in the *T. furgasoni* clade diverged 450,000 to 8.2 million years ago, *T. vorax* and nsp23 diverged 990,000 to 17.8 million years ago, and *T. pyriformis* and *T. leucophrys* diverged 1.1 to 20.6 million years ago. Of course, as mentioned above, it is possible these amicronucleates arose recently from uncollected or extinct micronucleate species. If it is the latter, then these amicronucleates may have survived their sexual ancestors because of macronuclear assortment, as discussed below.

**Figure 5:**
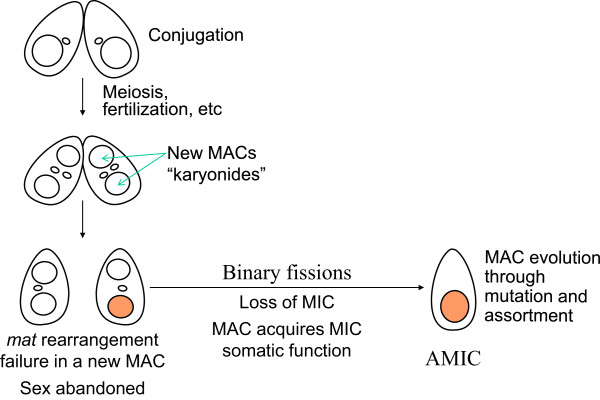
**Model for formation of amicronucleate *Tetrahymena*.** One or more errors of macronuclear development in a macronucleus result the failure to rearrange a functional *mat* gene, resulting in the loss of sex. Either simultaneously or through subsequent reproduction, the macronucleus acquires the essential somatic (oral) function of the MIC (see text), and eventually the MIC is lost. The resulting asexual amicronucleate is capable of independent evolution through mutation and macronuclear assortment.

Any hypothesis regarding the origin of amicronucleate tetrahymenas must account for two fundamental observations: their high frequency in nature and their inability to mate. In this respect it may be significant that most of the amicronucleates are in the “borealis” clade (Figure 4). Though not all breeding species have been examined, these two clades differ with respect to the mechanism of mating type determination [24,35]. In the “australis” clade mating type determination is “synclonal”, strictly determined by *mat* alleles inherited from each parent; all descendants of a conjugating pair have the same mating type because they have the same genotype. By contrast, mating type in the “borealis” clade is “karyonidal”, meaning that each of the four new macronuclei (karyonides) formed in conjugating pair is independently determined for mating type. In the karyonidal system, the micronuclear *mat* allele specifies a frequency distribution of mating types, one of which is chosen by a developing macronucleus. It is now known that for *T. thermophila* the mechanism involves sequence deletion and at least two recombination events during macronuclear development to form a functional *mat* locus from the inherited micronuclear gene [19]. This rearrangement results in a mating type region that contains two head to head genes, each encoding a transmembrane protein necessary for mating. The lack of mating in amicronucleates could be explained by developmental error in processing of either one of these genes. This hypothesis is modeled in Figure 5.

There are additional relevant observations. The first is the sole exceptional, viable amicronucleate *T.thermophila* which arose in the laboratory after chemical mutagenesis [13]. This vigorous strain (“pig”) does mate, albeit lethally, and perhaps more significantly, its macronucleus contains DNA sequences that are normally micronucleus limited [44], i.e., excised during macronuclear development. Perhaps these sequences or related errors of macronuclear development allowed survivorship. A second observation is that *T. thermophila* can be made nullisomic for any micronuclear chromosome (N = 5) and that all single and multiple nullisomics are viable and capable of conjugation yielding viable progeny [45]. This appears to rule out essential micronuclear sequences confined to a specific chromosome as necessary for cell viability. A third observation is that aging inbred strains of *T. thermophila* often lose micronuclear chromosomes, becoming severely hypodiploid [46]. These strains still conjugate vigorously and, though they cannot form functional gametic nuclei (they are functionally asexual), they nevertheless survive conjugation retaining the old macronucleus and emerging with a new, replacement micronucleus donated by the normal partner in a process called genomic exclusion [47]. After acquiring a new micronucleus such cells are capable of normal conjugation. If “pig” and genomic exclusion pathways are shared among tetrahymenas, then new amicronucleates capable of conjugation either die or receive new micronuclei upon first mating, leaving only amicronucleates incapable of conjugation in the population. The hypothesis in Figure 5 suggests that one or more errors during macronuclear development (perhaps epigenetically driven) result both in transfer of essential somatic micronuclear function(s) [1,17] to the macronucleus and in the non-mating phenotype. Subsequently, as the micronucleus accumulates genetic damage [10], it is lost, resulting in an amicronucleate cell.

The hypothesis presented in Figure 5 is potentially testable. Now that the *mat* gene has been identified [19], the most direct way is to ask whether the 12–13 kilobase pair *mat* locus is defective in amicronucleate *T. thermophila*. Preliminary experiments indicate that a *mat* locus is present and that the regions in which the recombination events occur are the correct size and do not contain frameshift mutations. However, given their large size, the complete genes have yet to be sequenced. An alternative hypothesis is that the lack of mating is due to permanent immaturity. Normally, *T. thermophila* are unable to mate until 40–60 fissions after conjugation, and wild *T. thermophila* are immature for at least 60, possibly 120 fissions [48]. Though genes resulting in early onset of maturity have been identified [49], the molecular mechanism of immaturity is not known.

The apparent success of *Tetrahymena* amicronucleates in natural habitats likely is related to their ability to continue to evolve by macronuclear assortment. As described in Background, many components of asexuality theory do not apply to ciliates or to *Tetrahymena* in particular. While Muller’s ratchet likely applies to micronuclei of all ciliates, there appear to be exceptions in its application to macronuclei, particularly *Tetrahymena. Tetrahymena* is the well known exception to the inability to maintain long term cultures of ciliates in the absence of sex [8]. Though clonal extinction may occur, cultures can be maintained, with full vigor, for decades. The *Tetrahymena* exception is attributable to the organization of the macronucleus that makes macronuclear assortment possible. No other ciliate assorts into phenotypically stable clones and at a rate inversely proportional to the number of gene copies (for reviews see [50,51]). The evolutionary origin of assortment is unknown, and what role the phenotypic diversity created by assortment has in the life cycle of micronucleate cells similarly is unknown. Any advantage assortment has to amicronucleate lineages (e.g., escape from Muller’s ratchet) must be a secondary consequence. Assortment occurs regardless of dominance relationships, exposing recessive alleles because dominant ones are lost as assortment proceeds. The macronucleus contains ~45 copies of each of the 180 macronuclear chromosomes [20]; these chromosomes are the assorting units [21] and they assort independently of each other. There is evidence that recombination also can occur between copies of individual chromosomes both during macronuclear development [52] and during binary fission [21]. Induced macronuclear mutations can assort [53,54], and assortment has been observed in both amicronucleate *T. pyriformis*[53] and micronucleate *T. canadensis*[55]. Thus, unlike genomes of asexual plants and animals, the macronuclear genome of *Tetrahymena* does not function as a single linkage group and therefore alleles at various loci can evolve independently. Muller’s ratchet likely does not apply, or is considerably slowed. A new neutral macronuclear mutation, though initially in the minority, can increase in frequency and eventually become fixed in a clonal lineage by assortment. If a mutation is deleterious, reducing reproductive success as it increases in number in an assorting lineage, selection should favor the reciprocal lineage which contains higher copies of the normal gene. Likewise, any beneficial mutation could succeed, and the resulting amicronucleate undergo adaptive evolution. Even low levels of recombination between linked genes within the macronucleus could result in combinations of favorable genes. This capability of independent evolution suggests that some *Tetrahymena* amicronucleates could be very ancient, perhaps surviving their micronuclear counterparts. Independent evolution of sexual and asexual strains suggests that amicronucleates might possess polymorphisms, both mitochondrial and macronuclear, not present among the micronucleate population. Further population studies are required to test this prediction.

Most of the named species of *Tetrahymena* with micronuclei are *bona fide* species as based on breeding tests, though there are a few, especially in the “americanis” clade, that need to be reexamined [25,33]. The use of molecular criteria to distinguish species has always been problematic when asexuals such as amicronucleates are concerned [23,56,57], and in any event the application is arbitrary. This paper used *cox*1 difference of 4% to declare an isolate as putative new species. As justified in Methods this value is a compromise which considers the range of pairwise differences between valid species and the upper limit of intraspecific variation. The 4% cut-off resulted in 19 putative new species, 18 if raised to 5%. In the larger project of which this survey is a part, there were 29 putative new species, 26 if the cutoff is raised to 6%. There are, however, valid species with *cox*1 differences of 5–6%; a cutoff of 4% is less likely to lump good species. The *cox*1 sequence of recently named amicronucleate species *T. aquasubterranea*[42] differs by 9.5% from its closest SSU relative nsp16, clearly supporting naming it as a new species. Because amicronucleates appear capable of evolution through macronuclear assortment, and indeed some amicronucleates appear to differ ecologically (e.g., NI/SU/RA/CO) or in food preferences (e.g., *T. vorax*) in addition to barcode sequences, designating them as species is reasonable. However, the naming of new species with detailed physical description becomes a formidable task given the apparent richness of *Tetrahymena* species. Moreover, though a goal of molecular taxonomy is to identify species without reference to living strains, the overlap of inter- and intraspecific *cox*1 sequence variation suggests that breeding tests on micronucleate species may be necessary in some instances. The threshold for amicronucleates as species will remain arbitrary. For protists there is no single satisfactory species concept [58].

## Conclusions

Amicronucleates of *Tetrahymena* are of multiple origins, independently arising from many micronucleate species. Some are of contemporary origin; others may be millions of years old. Amicronucleates are associated with 50% of *Tetrahymena* species and account for 25% of wild isolates, far in excess of what is observed in other ciliates. Given the limited geographical region sampled here and the abundance of amicronucleates in previous surveys, numerous additional amicronucleate species remain to be collected. Phylogenetically, most amicronucleates occur in the *Tetrahymena* clade in which each new macronucleus is independently determined for mating type by gene rearrangement. It is hypothesized that error(s) in this rearrangement process result in the inability to mate, followed by loss of the micronucleus. The abundance and apparent success of amicronucleates may be due to the unconventional nature of the macronucleus that allows it to escape the deleterious accumulation of mutations of Muller’s ratchet. Through macronuclear assortment and recombination, *Tetrahymena* amicronucleates are capable of evolution independent of sex.

## Availability of supporting data

The data set supporting the results of this article is available in the Dryad repository, http://dx.doi.org/10.5061/dryad.7t7d9[59].

## Abbreviations

ANF: Allegheny National Forest; EPA: Eastern Pennsylvania; WPA: Western Pennsylvania; LSU: Nuclear large ribosomal subunit rRNA; SSU: Nuclear small ribosomal subunit rRNA.

## Competing interests

The author declares no competing interest.

## Authors’ contributions

FPD did it all.

## Authors’ information

Professor, Department of Biological, Geological and Environmental Sciences, Cleveland State University, 2121 Euclid Avenue, Cleveland OH USA 44115.

## Supplementary Material

Additional file 1 **This Excel spreadsheet contains collecting information (date, site name, site type, state, latitude, longitude), species identification, SSU Genbank accession number for each amicronucleate species, and *cox*** **1 accession numbers for each haplotype.** It also contains similar data for relevant micronucleate isolates. Click here for file
